# Correction: BWGS: A R package for genomic selection and its application to a wheat breeding programme

**DOI:** 10.1371/journal.pone.0232422

**Published:** 2020-04-23

**Authors:** The PLOS ONE Staff

## Notice of Republication

This article was republished on April 8, 2020, to correct an erroneous unicode character in the Materials and methods section. The publisher apologies for the error. Please download this article again to view the correct version.
